# Menaquinone-7 Supplementation to Reduce Vascular Calcification in Patients with Coronary Artery Disease: Rationale and Study Protocol (VitaK-CAC Trial)

**DOI:** 10.3390/nu7115443

**Published:** 2015-10-28

**Authors:** Liv M. Vossen, Leon J. Schurgers, Bernard J. van Varik, Bas L. J. H. Kietselaer, Cees Vermeer, Johannes G. Meeder, Braim M. Rahel, Yvonne J. M. van Cauteren, Ge A. Hoffland, Roger J. M. W. Rennenberg, Koen D. Reesink, Peter W. de Leeuw, Abraham A. Kroon

**Affiliations:** 1 Department of Internal Medicine, Maastricht University Medical Centre (MUMC+), Maastricht 6229HX, The Netherlands; b.vanvarik@maastrichtuniversity.nl (B.J.V.); r.rennenberg@mumc.nl (R.J.M.W.R.); p.deleeuw@maastrichtuniversity.nl (P.W.L.); aa.kroon@mumc.nl (A.A.K.); 2 Department of Internal Medicine, Zuyderland Medical Centre, Sittard 6162BG, The Netherlands; 3 Department of Biochemistry, Cardiovascular Research Institute Maastricht (CARIM), University of Maastricht, Maastricht 6229ER, The Netherlands; l.schurgers@maastrichtuniversity.nl (L.J.S.); k.reesink@maastrichtuniversity.nl (K.D.R.); 4 Departments of Cardiology and Radiology, Maastricht University Medical Centre (MUMC+), Maastricht 6229HX, The Netherlands; b.kietselaer@mumc.nl; 5 R&D Group VitaK, Maastricht University, Maastricht 6229EV, The Netherlands; c.vermeer@vitak.com; 6 Department of Cardiology, VieCuri Medical Centre, Venlo 5912 BL, The Netherlands; jmeeder@viecuri.nl (J.G.M.); brahel@viecuri.nl (B.M.R.); yvonne.cauteren@mumc.nl (Y.J.M.C.); 7 Department of Radiology, VieCuri Medical Centre, Venlo 5912BL, The Netherlands; ghoffland@viecuri.nl

**Keywords:** vascular calcification, coronary artery calcification, matrix gla protein, vitamin K2, menaquinone-7

## Abstract

Coronary artery calcification (CAC) develops early in the pathogenesis of atherosclerosis and is a strong and independent predictor of cardiovascular disease (CVD). Arterial calcification is caused by an imbalance in calcification regulatory mechanisms. An important inhibitor of calcification is vitamin K-dependent matrix Gla protein (MGP). Both preclinical and clinical studies have shown that inhibition of the vitamin K-cycle by vitamin K antagonists (VKA) results in elevated uncarboxylated MGP (ucMGP) and subsequently in extensive arterial calcification. This led us to hypothesize that vitamin K supplementation may slow down the progression of calcification. To test this, we designed the VitaK-CAC trial which analyses effects of menaquinone-7 (MK-7) supplementation on progression of CAC. The trial is a double-blind, randomized, placebo-controlled trial including patients with coronary artery disease (CAD). Patients with a baseline Agatston CAC-score between 50 and 400 will be randomized to an intervention-group (360 microgram MK-7) or a placebo group. Treatment duration will be 24 months. The primary endpoint is the difference in CAC-score progression between both groups. Secondary endpoints include changes in arterial structure and function, and associations with biomarkers. We hypothesize that treatment with MK-7 will slow down or arrest the progression of CAC and that this trial may lead to a treatment option for vascular calcification and subsequent CVD.

## 1. Introduction and Rationale

Coronary artery calcification (CAC) develops early in the pathogenesis of atherosclerosis [1] and is a strong and independent risk marker of cardiovascular complications [2,3]. Moreover, annual changes in CAC-score are thought to reflect changes in atherosclerotic plaque burden [1,3]. Vascular calcification is not merely a passive phenomenon but rather, is caused by an imbalance between the mechanisms that promote and inhibit the deposition of calcium in the vascular wall. In this regard the vitamin K dependent Matrix Gla protein (MGP) plays an important role [3,4,5,6,7] as an inhibitor of soft tissue calcification, as was first shown in MGP-deficient mice [4]. Inhibition of the vitamin-K-cycle by vitamin K antagonists (VKA) results in the accumulation of uncarboxylated MGP which is biologically inactive. This is associated with extensive arterial calcification in experimental animals [5,8]. In line with the experimental data, humans on VKA treatment also tend to have more aortic and valve calcification in comparison to patients not on anticoagulant therapy [9]. Furthermore, observational studies show that long-term use of VKA is associated with both increased extra-coronary vascular calcification [10] and increased coronary calcification [11]. Because VKA induce vascular calcification, vitamin K supplementation presents an attractive treatment option to reduce vascular calcifications [9,12,13]. Indeed, in rats, dietary supplementation with high doses of either phylloquinone (vitamin K1) or menaquinone-4 (vitamin K2) resulted in the regression of post-warfarin-induced arterial calcifications [14,15]. In mice, co-administration of low-dose MK-4 in the concurrent warfarin plus phylloquinone model of vascular calcification reduced the development of calcification [16]. Note that in this animal model of warfarin-induced vascular calcification, the concurrent administration of phylloquinone is needed to support hepatic synthesis of coagulation factors but does not itself prevent vascular calcification while warfarin is still being administered. Observational studies in humans show an inverse relationship between menaquinone intake and CAC in healthy elderly [17,18]. However, phylloquinone supplementation itself was shown to slow the progression of CAC and had a beneficial effect on vascular stiffness in healthy adults with coronary artery calcification after 3 years of follow-up [19,20,21]. Dalmeijer *et al.* performed a randomized, double blind, placebo controlled trial to investigate the effect of menaquinone-7 (MK-7) supplementation on MGP species and found a dose-dependent decrease of dephospho-uncarboxylated MGP (dp-ucMGP) concentrations [22]. Furthermore, MK-7 improves arterial stiffness and elastic properties of the carotid artery [23]. Altogether, this data suggests that vitamin K administration may have beneficial effects on the vasculature. So far, however, the effect of MK-7 supplementation on CAC and its long-term progression has not been studied in humans in a randomized clinical trial (RCT). Therefore, we designed the VitaK-CAC trial to analyze the effect of MK-7 supplementation in comparison to placebo on the annual progression of CAC. We hypothesize that treatment with MK-7 will slow down or arrest the progression of CAC in comparison to placebo and that MK-7 supplementation may lead to a treatment option for vascular calcification and cardiovascular disease.

Our proposed RCT has several highly innovative features. First it will be the first RCT to study the effect of nutritional supplementation with MK-7 on the progression of arterial calcification using state of the art techniques for the quantification of calcification in atherosclerotic lesions in patients with pre-existing CAC. The mainstay of the assessment of CAC progression will be multi-slice computed tomography in addition to measurements of arterial stiffness used in previous studies of MK-7 [23]. The only other comparable RCT using Computed Tomography (CT) scans showed that daily supplementation with 500 μg phylloquinone (high dietary range) reduced the progression of CAC in older people by 6% [19]. From the animal and human studies outlined above [12,16,17,18,24] we hypothesize that MK-7 should be more effective than phylloquinone in ameliorating the progression of CAC. One likely reason for the greater effectiveness of MK-7 is that its rate of clearance from the human circulatory system is much longer than that for phylloquinone and enhances the degree of gamma-carboxylation of Gla proteins in extrahepatic tissues such as bone and arteries [12]. Finally we should emphasize that the proposed daily amounts of 360 μg MK-7 are within the nutritional range of certain diets. For example the MK-7 content of Japanese fermented food natto is approximately 1000 μg/100 g in a highly bioactive form for gamma-carboxylation of hepatic and extrahepatic Gla proteins [12,25].

## 2. The Vitamin K—Coronary Artery Calcification (VitaK-CAC) Study

### 2.1. Trial Objectives

The primary objective of our trial is to assess whether oral MK-7 supplementation will slow down the rate of CAC progression after 12 and 24 months in patients with pre-existing CAC in comparison to treatment with placebo. Secondary endpoints include CT angiographically defined plaque composition alterations, and changes in parameters of arterial stiffness, extra-coronary atherosclerosis and biomarkers (Table 1).

**Table 1:** Secondary endpoints.

Changes in plaque morphology of existing atherosclerotic lesions
Incidence of new calcified atherosclerotic lesions
Changes in arterial structure and function
*Carotid-femoral pulse wave velocity (cfPWV)*
*Pulse-waveform and central aortic blood pressure (CABP)*
*Common carotid artery intima media thickness (cIMT)*
*Common carotid artery distensibility coefficient (DC)*
Biochemical associations between CAC progression
*Circulating matrix Gla protein (MGP) species with different phosphorylation and carboxylation forms*
*Osteocalcin (OC)*
*Lipid profile (Total cholesterol, Low-Density Lipoprotein-cholesterol (LDL), High-Density Lipoprotein-cholesterol (HDL) and Triglycerides)*
*Glucose status (Fasting glucose)*
*Calcium metabolism (Calcium, Albumine, Phosphate and Parathyroid Hormone)*
*Kidney function (Creatinine)*
*Prothrombin time-International normalized ratio (PT-INR)*

### 2.2. Patient Recruitment

The VitaK-CAC trial is a double-blind, randomized, placebo-controlled trial in two centers. Eligible patients suspected of having coronary artery disease (CAD) and who are scanned by 128-slice multiple detector CT (MDCT) to assess the presence of coronary atherosclerotic plaques and CAC will be identified from the outpatient clinic of the departments of Cardiology. Patients with a baseline Agatston CAC-score between 50 and 400 will be recruited and randomized into two groups. This Agatston score corresponds with a moderately increased risk of cardiovascular events or death [2].

The study has been approved by the Medical Ethics Committee (MEC) of the Maastricht University Medical Center, Maastricht, the Netherlands. The study complies with the Declaration of Helsinki. All patients have to give written informed consent.

The VitaK-CAC trial is registered at clinicaltrials.gov as NCT01002157.

### 2.3. Inclusion and Exclusion Criteria

We include all patients older than 18 years with a baseline CAC-score (Agatston) between 50 and 400. Patients who meet any of the exclusion criteria (Table 2) will be excluded. Specifically, patients with chronic kidney disease (CKD) will be excluded because of differences in pathophysiology of vascular calcification and higher prevalence of cardiovascular death [26]. Disorders of calcium and phosphate metabolism and a high prevalence of subclinical vitamin K deficiency in this population contribute to the higher rate of vascular calcification and mortality [14].

**Table 2:** Exclusion criteria.

Baseline-scan of insufficient quality (due to the presence of motion artefacts, breathing artefacts or high noise-levels)
Heart rate greater than 70 beats per min during first scan because of impaired scan quality
Chronic or paroxysmal atrial fibrillation
Presence or scheduled bypass-grafting in more than one coronary artery
Presence or scheduled coronary revascularization procedure (stent-placement > 1 coronary artery)
History of myocardial infarction or stroke < 6 months before coronary Coronary Tomography (CT)
Presence of diabetes mellitus type 1
Known kidney disease or an estimated Glomerular Filtration Rate (eGFR) < 60 mL/min/1.73 m^2^, calculated by the MDRD-formula
Malignant disease (exception: treated basal-cell or squamous cell carcinoma)
Use of Vitamin K antagonists
A life-expectancy < 2 years
Pregnancy or wish to become pregnant in the near future

### 2.4. Measurements

#### 2.4.1. Interviewing, Physical Examination and Blood-Pressure Measurement

At baseline, information on medical history, lifestyle factors (smoking, alcohol consumption, physical activity and dietary habits) and use of medication will be assessed. Furthermore, height, weight and waist-hip circumference will be measured. Arterial blood pressures (systolic, diastolic and mean) will be measured on both arms with a validated electronic (oscillometric) blood-pressure measurement device (Datascope Accutorr Plus, Soma Technology, Paramus, NJ, USA). Before measurement, subject will be seated for at least 5 min. At every study visit, drug adherence and any co-medication will be recorded.

#### 2.4.2. Multi-Slice Computed Tomography

Scans will be performed using a dual-source CT-scanner (Somatom Definition Flash, Siemens Medical Solutions, Forchheim, Germany). In line with routine clinical procedures, patients can be premedicated with beta blocking agents to achieve a stable heart rate and/or sublingual nitrates to ensure vasodilatation. First, a native scan is performed using 120 kV and 3 mm slice thickness to determine the calcium score according to the Agatston method [27]. Subsequently, a 20 mL contrast test bolus will be injected to assess the time to peak in the ascending aorta. Coronary computed tomography angiography (CCTA) will be performed using 80–100 mL of contrast agent (Ultravist 300; Bayer Pharma AG, Berlin, Germany), which is injected into an antecubital vein at a rate of 5.2–7.4 mL/s followed by 60 mL intravenous saline (6.0 mL/s) using a dual-head power injector (Medrad Inc., Pittsburgh, PA, USA). A prospectively gated high pitch spiral “flash” protocol will be used in patients with a stable heart rate <60 beats per minute (bpm). In patients with a stable heart rate between 60 and 90 bpm, a prospectively gated axial “adaptive sequence” protocol is used. In patients with a heart rate > 90 bpm or in case of an irregular heart rhythm (because of new diagnosed atrial fibrillation or ventricular extrasystoles), a retrospectively gated “helical” protocol with dose modulation will be used. Data acquisition parameters are 2 × 128 × 0.6 mm slice collimation, a gantry rotation time of 280 millisecond (ms) and a tube voltage of 100 or 120 kV depending on patients’ height and weight.

The assessment will be performed using the source images on the provided software (Syngo CT 2010A, Siemens, Forchheim, Germany). The coronary artery tree will be analyzed for the presence and severity of CAD, according to the classification of the American Heart Association 16-segment model [28]. Coronary plaques are defined as visible structures within or adjacent to the coronary artery lumen, which can be clearly distinguished from the vessel lumen and the surrounding pericardial tissue. Quantification of coronary plaque components is done via semi-automated analysis as has been described before [29]. Scans are analyzed independently by a cardiologist and a radiologist, both experienced in the assessment of CCTA. In case of disagreement, consensus is reached by discussion.

#### 2.4.3. Measurement of the Central Aortic Blood Pressure (CABP)

Measurement of the CABP is based on pulse wave analysis using a Sphygmocor device (SphygmoCor, Atcor Medical, Sydney, Australia). The waveform is recorded using a pen-shaped transducer that is placed on the skin of the patient's wrist overlying the radial artery (application tonometry). Using brachial mean arterial pressure and diastolic blood pressure for calibration and a transfer function, the CABP is estimated by the Sphygmocor internal algorithms.

#### 2.4.4. Measurement of Pulse Wave Velocity (PWV)

The carotid-to-femoral and carotid-to-radial pulse wave velocities (cfPWV and crPWV) will be determined according to recent guidelines by using tonometry (Complior, Artech Medical, Patin, France) [30]. With the participant in supine position, sensors will be placed on the skin over the right common carotid artery, right radial artery, and right femoral artery. After obtaining simultaneous waveforms of sufficient quality, two consecutive series of 10 to 15 waveforms will be recorded for derivation of transit times. The transit time will be determined using the intersecting tangent algorithm. The distances between the sensors will be measured to calculate the cfPWV and crPWV. The median of two consecutive cfPWV and crPWV recordings will be used in the analysis.

#### 2.4.5. Measurement of the Carotid Intima Media Thickness (cIMT)

The carotid Intima Media Thickness is measured using a vascular ultrasound scanner equipped with the ArtLab wall-track system (Esaote/Pie Medical, Maastricht, the Netherlands). With the participant lying supine, both rights and left Common Carotid Arteries (CCA) are identified on a longitudinal ultra-sonographic image. During ultrasound measurements, a double line pattern on both walls of the CCA is detected in real-time, consisting of the echoes of the lumen-intima transition and media-adventitia transition (automated radiofrequency-based IMT). The cIMT measurement will be done 10 mm proximal to the carotid bulb and is determined on the far wall of the CCA as the distance between these two lines in micrometers (spatial average). Images will be taken at four different angles (90°, 120°, 150° and 180° for the right carotid artery, and 180°, 210°, 240° and 270° for the left carotid artery). The median diameter and IMT of the measurements will be used in the analysis. The cIMT measurements will we performed by certified operators. Because of the radiofrequency-based IMT measurement we don’t expect interobserver nor intra-observer variability [31]. The reproducibility will be calculated.

#### 2.4.6. Measurement of the Common Carotid Artery Distensibility Coefficient (DC)

The CCA distensibility coefficient (DC) is measured using a vascular ultrasound scanner equipped with an ArtLab wall-track system (Esaote Europe, Maastricht, the Netherlands). The DC is calculated as follows: $DC\;=\;\frac{2\Delta D\;\times \;D\;+\;\Delta D^{2}}{PP\;\times \;D^{2}}$, in (10^−3^·kPa^−1^), where D is the arterial (diastolic) diameter, ∆D is the distension, and PP is the brachial pulse pressure (calculated as systolic minus diastolic blood pressure). The median DC of two measurements will be used in the analysis.

#### 2.4.7. Laboratory Assessment

Fasting blood samples will be obtained for routine biochemical and hematological measurements and specific laboratory variables (see Table 3). In addition, 7 mL of blood will be stored for future research. MGP will be quantified using an automated method available on the market (supplied by IDS, Boldon, UK). OCN will be measured using Gla-OC and Glu-OC ELISAs from Takara (Shiga, Japan).

**Table 3:** Laboratory assessment.

Routine Laboratory Variables	Specific Laboratory Variables
Total cholesterol	MGP OCN
LDL-cholesterol	
HDL-cholesterol	
Triglycerides	
Creatinine	
Glucose	
Albumin	
Calcium	
Phosphate	
Coagulation function (PT-INR)	

### 2.5. Treatment Schedule

**Figure 1:**
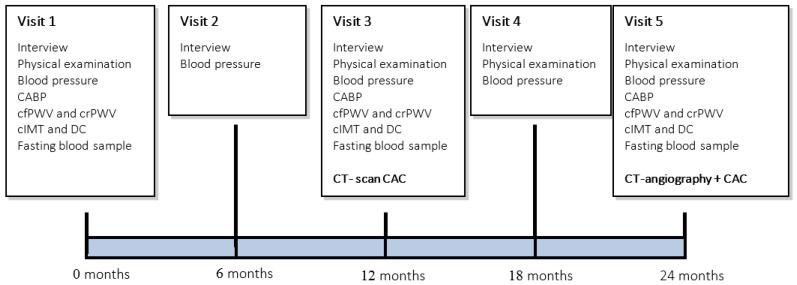
Trial design.

Treatment of both groups will last for 24 months. During this time participants will visit our research unit five times, at 6-month intervals (Figure 1). At every study visit we will perform an interview and blood pressure measurements. During the first visit, at 12, and at 24 months of follow-up, measurements of CABP, cfPWV, crPWV, cIMT and DC are scheduled. In addition, fasting blood-samples will be drawn at 0, 12, and 24 months. We will repeat CT-scans of the heart and coronary arteries at 12 and 24 months of follow-up. At 12 months only a non-contrast CT scan will be performed to obtain a calcium score. At 24 months a CT-angiography will be performed, using the same acquisition protocol and reconstruction techniques as the initial CT scan (See Figure 1).

### 2.6. Vitamin K Product

Subjects in the intervention group will receive a once-daily oral tablet of 360 micrograms of MK-7. In our study we use a pure synthetic MK-7 provided by NattoPharma ASA (Hovik, Norway). The choice for MK-7 as K-vitamer is based on the longer half-life and extra-hepatic tissue distribution as compared to other vitamin K-forms [12,24]. The chosen dose in this trial was established in a dose-finding study, in which the effect of increasing doses of MK-7 on OCN and MGP-carboxylation was monitored. A daily dose of 360 μg MK-7 most effectively reduced the amount of non-functional MGP [22,32].

MK-7 is well tolerated and does not cause a hypercoagulable state [33]. There are no reported adverse effects associated with the use of MK-7.

### 2.7. Randomization Procedure

If the patient is eligible and willing to participate in the study, the patient will be randomly assigned to the MK-7 or placebo group. Subjects will be randomized after stratification for age, sex, BMI, statin- and bisphosphonate-use using the minimization technique [34]. An independent investigator who is not involved in the coordination or analysis of this study, will randomize consecutive patients using custom-made computer software. The randomization-list will be stored in a secure location and will not be accessible to the investigators during the study.

### 2.8. Statistical Analysis

The main outcome parameters (CAC-score) and CAC-score progression will be presented as continuous variables. In addition, the amount of CAC-score progression (in both Agatston-score and Mass-score) will be dichotomized (rapid progression and slow progression) using a cut-off of an annual progression of 15% [33].

Data will be analyzed based on the intention-to-treat principle. To adjust for potential confounders (age, gender, BMI, blood-pressure, smoking, cholesterol, glucose and medication use), multiple analysis of variance will be performed. For continuous variables, multivariable linear regression will be applied. For longitudinal data analysis (*i.e.*, progression between baseline, 12 months, and 24 months of follow-up) Generalized Estimating Equations (GEE) analysis will be applied to adjust for multiple testing. Additional statistical analyses will be performed as appropriate (e.g., Hazzard ratio calculation). All statistical analysis will be performed using the statistical package SPSS 22 (IBM Corp, Armonk, New York, USA). The statistical tests will be performed using a two-sided significance level of 5%.

### 2.9. Sample Size

The mean annual CAC-progression reported in literature ranges from 24% to 51% and has a large inter-individual variation depending on many factors such as the baseline CAC-score, medical history, medication-use, body-mass index, scanner type and manufacturer. The standard deviation in CAC-score progression in a population comparable to our intended population is reported to range from 21% to 36% [33]. Since we use strict inclusion and exclusion criteria and use a 128-slice MDCT with better reproducibility of measurements, we estimate the standard deviation of CAC-progression to be 25%. We consider an absolute difference of 15% in CAC-progression between the two treatment groups a significant effect [33]. To test this difference with a statistical power of 90% (alpha 0.05) we require 59 patients per group. Based on analysis of available data from our center, we estimate a patient drop-out of approximately 35%. This drop-out may have various reasons such as patients requiring coronary artery stenting over the course of the study, occurrence of cardiovascular events such as myocardial infarction requiring percutaneous coronary intervention (PCI) or death, development of exclusion criteria, or subjects no longer willing to participate in the study. Therefore, to obtain sufficient statistical power to demonstrate a possible treatment-effect, we require 90 patients per group for a total number of 180 patients.

### 2.10. Organization

#### 2.10.1. Data Safety Monitoring Board (DSMB)

An independent DSMB will be established to perform ongoing safety surveillance and to perform interim analyses on the safety data. None of the DSMB-members are directly or indirectly involved in the coordination, execution or analysis of the proposed study.

The DSMB’s primary task is to monitor the safety of participants. Interim analysis will be performed to assess whether a treatment-group has relatively more adverse events than the other. The DSMB will assess the number of cardiovascular events (myocardial infarction, coronary revascularization procedures, and cardiovascular deaths) and the number of adverse reactions/side effects as reported by the study-participants at every interim analysis.

Furthermore, the DSMB will calculate the rate of progression of vascular calcification in both groups. If there is a reason for concern, the DSMB can advise to interrupt the study for further analysis or terminate the study. This will be discussed in a meeting with the investigators and DSMB.

The investigator will inform the subjects and the reviewing accredited MEC if anything occurs, on the basis of which it appears that the disadvantages of participation may be significantly greater than was foreseen in the research proposal.

#### 2.10.2. Adverse and Serious Adverse Events (AE and SAE)

Adverse events are defined as any undesirable experience occurring to a subject during a clinical trial, whether or not they are considered related to the investigational drug. All adverse events are recorded. An abnormal laboratory result will not be considered as an adverse event except where it is indicative of disease and/or organ toxicity. Details of all adverse events reported spontaneously by subjects or observed by the investigator or medical staff will be recorded. All SAEs will be reported to the accredited MEC that approved the protocol, according to the local requirements.

#### 2.10.3. Suspected Unexpected Serious Adverse Reactions (SUSAR)

Adverse reactions are all untoward and unintended responses to an investigational product related to any dose administered. Unexpected adverse reactions are adverse reactions, of a nature or severity that is not consistent with the applicable product information. The investigator will report all SUSARs to the MEC. In case of a SUSAR, the randomization-code will be broken for the individual patient only so blinding of treatment allocation can be maintained for the remaining study subjects. The treatment allocation of that individual patient will be reported to the investigators.

#### 2.10.4. Premature Termination of the Study

The study can be terminated prematurely if the number of SAEs or the rate of vascular calcification is significantly higher in the treatment group *versus* the placebo group.

The study will be terminated if a causal relation between MK-7 treatment and the adverse events or progression of vascular calcification is highly suspected. If the study is terminated prematurely, all subjects will be informed about their results and the reason why the study is terminated.

#### 2.10.5. Withdrawal of Individual Subjects

Subjects can leave the study at any time for any reason if they wish to do so, without any consequences. The investigator can decide to withdraw a subject from the study for urgent medical reasons or in case of demonstrable poor adherence to the study medication. This is assessed by interview and pill-count. If subjects are required to take vitamin-K antagonists (coumarins) during the course of the study (for example due to Deep-Venous Thrombosis or Pulmonary Embolism) they will be withdrawn from the study.

#### 2.10.6. Participating Centers

The Maastricht University Medical Centre (MUMC) is the initiating center of the VitaK-CAC trial and the VieCuri Medical Centre is participating. The principal investigator is Abraham A. Kroon, internist at the MUMC.

## 3. Concluding Remarks

In conclusion, the VitaK-CAC trial will study the effect of menaquinone-7 supplementation on progression of CAC in a randomized, placebo-controlled trial. We hypothesize that MK-7 supplementation will slow down the progression of CAC in patients with CAD. So far, no treatment options are available for vascular calcification, and this trial may lead to a treatment option for vascular calcification and cardiovascular disease.

The result of this study will be expected at the end of 2017.
